# Circadian Rhythms of Clock Genes After Transplantation of Mesenchymal Stem Cells with Type 2 Diabetes Mellitus

**DOI:** 10.3390/ijms252313145

**Published:** 2024-12-06

**Authors:** Michiko Horiguchi, Kenichi Yoshihara, Kenji Watanabe, Yuya Tsurudome, Yoichi Mizukami, Kentaro Ushijima

**Affiliations:** 1 Division of Pharmaceutics, Faculty of Pharmaceutical Sciences, Sanyo-Onoda City University, Yamaguchi 756-0884, Japan; 2 Institute of Gene Research, Yamaguchi University Science Research Center, Yamaguchi 753-0841, Japan

**Keywords:** circadian rhythm, clock genes, mesenchymal stem cells (MSCs), type 2 diabetes mellitus, MTATP8P1, NDUFA7_2

## Abstract

Regenerative therapy involving stem cell transplantation has become an option for the radical treatment of diabetes mellitus. Disruption in the clock genes of stem cells affects the homeostasis of transplanted tissues. We examined the circadian rhythm of genes in transplanted adipose-derived mesenchymal stem cells derived from a patient with type 2 diabetes mellitus (T2DM-ADSC). The clock genes (PER2, CLOCK1, CRY1, and ARNTL[BMAL1]) exhibited similar daily fluctuations in phase and amplitude between a group transplanted with adipose-derived mesenchymal stem cells derived from a healthy individual (N-ADSC) and a group transplanted with T2DM-ADSC. The findings demonstrated that clock genes in stem cells are synchronized with those in living organisms. Next-generation sequencing was then employed to categorize genes that exhibited variation in expression between N-ADSC and T2DM-ADSC. MTATP8P1 and NDUFA7_2 gene expression was significantly reduced at two time points (ZT6 and ZT18), and daily fluctuations were lost. The present study reports, for the first time, that the circadian rhythms of MTATP8P1 and NDUFA7_2, genes involved in mitochondrial processes, are altered in T2DM-ADSC.

## 1. Introduction

Diabetes mellitus is a disease characterized by long-term abnormalities in sugar metabolism due to the decreased secretion of insulin and increased insulin resistance [1,2]. According to the 10th Edition of the International Diabetes Federation Diabetes Atlas, there were 537 million patients (aged 20–79) with diabetes worldwide in 2021. As the disease progresses, complications of diabetic retinopathy (e.g., sight loss) begin to occur [3,4]. Improvements in lifestyle habits and insulin replacement therapy are typical measures utilized for the treatment of diabetes [5]. In recent years, regenerative therapy using stem cell transplantation has become an option for radical treatment in this setting [6,7,8].

Regenerative therapy for diabetes involves the use of various stem cells [9]. For example, mesenchymal stem cells (MSCs), which can be stably supplied, are transplanted in an attempt to promote the secretion of proliferative and tissue regeneration factors [10]. MSCs can be obtained from diverse tissues [11,12]. Adipose-derived mesenchymal stem cells (ADSCs) are characterized by greater immunosuppressive ability compared to those derived from bone marrow [13]. Reports indicate that ADSCs produce large quantities of vascular endothelial growth factor (VEGF) and hepatocyte growth factor (HGF) [14]. Accordingly, ADSCs have attracted attention as an excellent cell material for regenerative therapy in patients with severe, rapidly progressing diabetes [15]. Efficiency is the key to effective regenerative therapy via ADSC transplantation. However, it remains unclear whether autologous ADSCs derived from patients with underlying diabetes can achieve similar engraftment to that of ADSCs extracted from healthy individuals.

We previously evaluated N-ADSCs (ADSCs derived from a healthy individual), T1DM-ADSCs (ADSCs derived from a patient with type 1 diabetes mellitus [T1DM]), and T2DM-ADSCs (ADSCs derived from a patient with type 2 diabetes mellitus [T2DM]) [16,17,18,19]. We demonstrated that the transplantation efficiency of T2DM-ADSCs was lower than that of N-ADSCs [19]. Subsequently, we performed an exhaustive mRNA analysis using next-generation sequencing and identified glucose-6-phosphatase catalytic subunit 3 (G6PC3) and insulin-like growth factor 1 (IGF1) as genes responsible for the difference in transplantation efficiency [19]. Moreover, we revealed that the reduced transplantation efficiency of T2DM-ADSCs was improved by knocking down G6PC3 and IGF1 [19]. These results clarified the characteristics of T2DM-ADSCs. However, a post-transplantation functional evaluation has not previously been conducted.

Previously reported clock genes include the period circadian regulator (PER), clock (CLOCK), and cryptochrome (CRY) [20]. These genes are indispensable for maintaining homeostasis [20]. Clock genes exist in most cells in the human body, and the central clock controlling these genes is situated in the suprachiasmatic nucleus (SCN) [21]. In preceding studies, we analyzed the functions of clock genes using models of diabetes and obesity [22,23]. It has been suggested that the circadian clock has a significant effect on stem cell differentiation, and a relationship between the circadian clock and the fate determination of mesenchymal stem cells in the skeletal system has been reported [24]. The underlying mechanisms include hormonal signals and the activation and repression of various transcription factors under circadian regulation. The clock interacts with epigenetic modifiers and non-coding RNAs and is also involved in chromatin remodeling [24]. Furthermore, it has been suggested that stem cell aging and the perturbation of circadian rhythms are closely intertwined [25]. The origin of circadian rhythms in stem cells and their functions in differentiated cells and organs have also been elucidated [26]. Therefore, it is important to monitor the circadian rhythms of clock genes and the genes regulated by clock genes after the transplantation of stem cells.

The purpose of this study is to determine the genetic circadian rhythm after the transplantation of stem cells derived from T2DM patients. In particular, we clarify whether the circadian rhythms of *PER1*, *PER2*, *PER3*, *CLOCK1*, *CLOCK2*, *CLOCK3*, *CLOCK4*, *CRY1*, *CRY2*, and *BMAL1* are retained in the graft after the transplantation of stem cells derived from T2DM patients. Then, we identify the genes that show circadian rhythm variation in the T2DM-ADSC transplant group.

## 2. Results

**N-ADSCs and T2DM-ADSCs exhibited similar circadian rhythms of clock genes in terms of phase and amplitude after transplantation**

**Figure 1:**
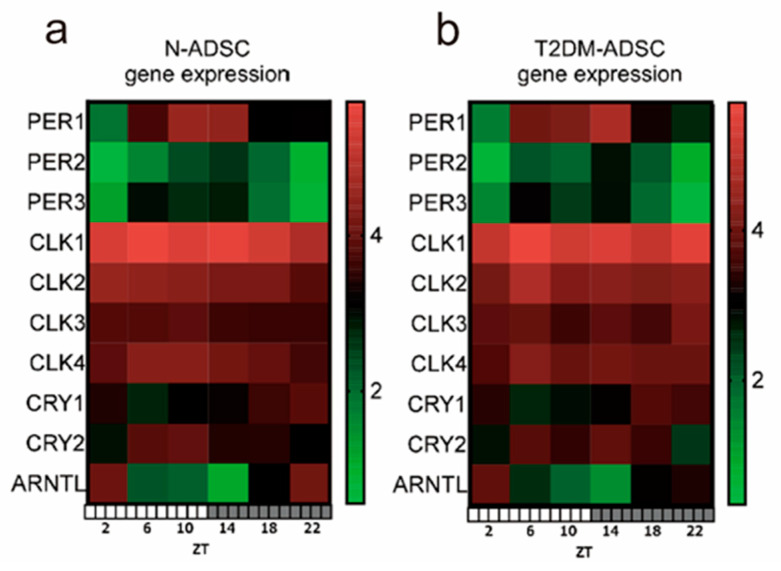
**The measurement of 24 h clock gene expression via next-generation sequencing after the transplantation of adipose-derived mesenchymal stem cells derived from a healthy individual and a patient with type 2 diabetes mellitus.** (**a**) Clock gene expression in adipose-derived mesenchymal stem cell transplants derived from a healthy individual. (**b**) Clock gene expression in adipose-derived mesenchymal stem cell transplants derived from a patient with type 2 diabetes mellitus. Abbreviations in figure: N-ADSC, adipose-derived mesenchymal stem cells derived from a healthy individual; T2DM-ADSC, adipose-derived mesenchymal stem cells derived from a patient with type 2 diabetes mellitus; ZT, Zeitgeber time.

The results of next-generation sequencing revealed that the N-ADSC transplants exhibited daily fluctuations in the clock genes *PER1*, *PER2*, *PER3*, *CRY1*, *CRY2*, and aryl hydrocarbon receptor nuclear translocator like (*ARNTL*) (basic helix-loop-helix ARNT like 1 [*BMAL1*]). The circadian rhythm was observed with *PER1* peaking at ZT10 and ZT14, *PER2* peaking at ZT14, and *PER3* peaking at ZT6. *CRY1* showed a peak at ZT22, and *CRY2* showed a circadian rhythm with peaks at ZT22 and ZT2, as did *ARNTL(BMAL1)*. In contrast, daily fluctuations were not noted in the clock genes *CLOCK1*, *CLOCK2*, *CLOCK3*, and *CLOCK4* (Figure 1a,b).

**Figure 2:**
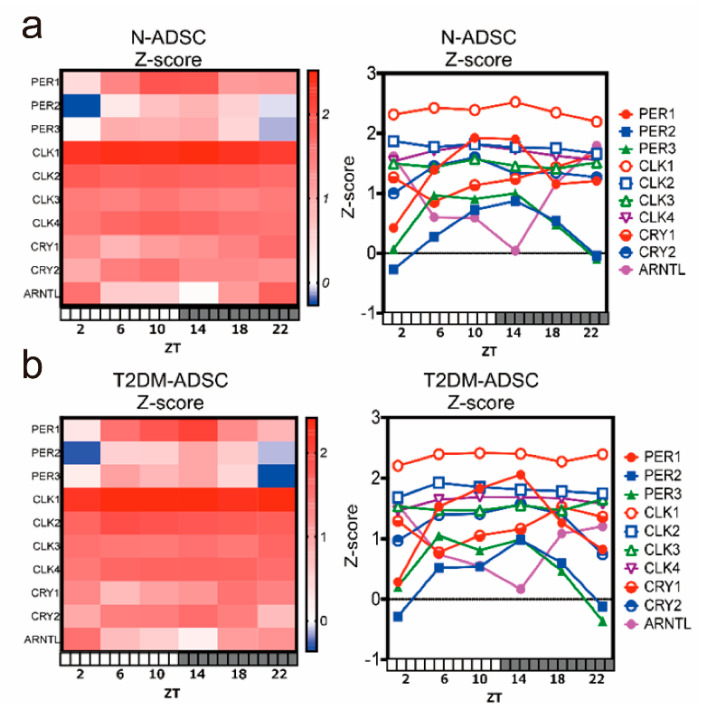
**The 24 h clock gene expression was measured via next-generation sequencing, displaying the Z-score.** (**a**) Clock gene expression score in adipose-derived mesenchymal stem cells derived from transplants from a healthy individual. (**b**) Clock gene expression score in adipose-derived mesenchymal stem cells derived from transplants from a patient with type 2 diabetes mellitus.

Similarly, the T2DM-ADSCs showed daily fluctuations in *PER1*, *PER2*, *PER3*, *CRY1*, *CRY2*, and *ARNTL* (*BMAL1*), whereas such fluctuations were not observed in *CLOCK1*, *CLOCK2*, *CLOCK3*, and *CLOCK4* (Figure 2a). In the Z-score display of next-generation sequencing, daily fluctuations were noted in *PER1*, *PER2*, *PER3*, *CRY1*, *CRY2*, and *ARNTL* (*BMAL1*) in both the N-ADSCs (Figure 2a) and T2DM-ADSCs (Figure 2b).

**Figure 3:**
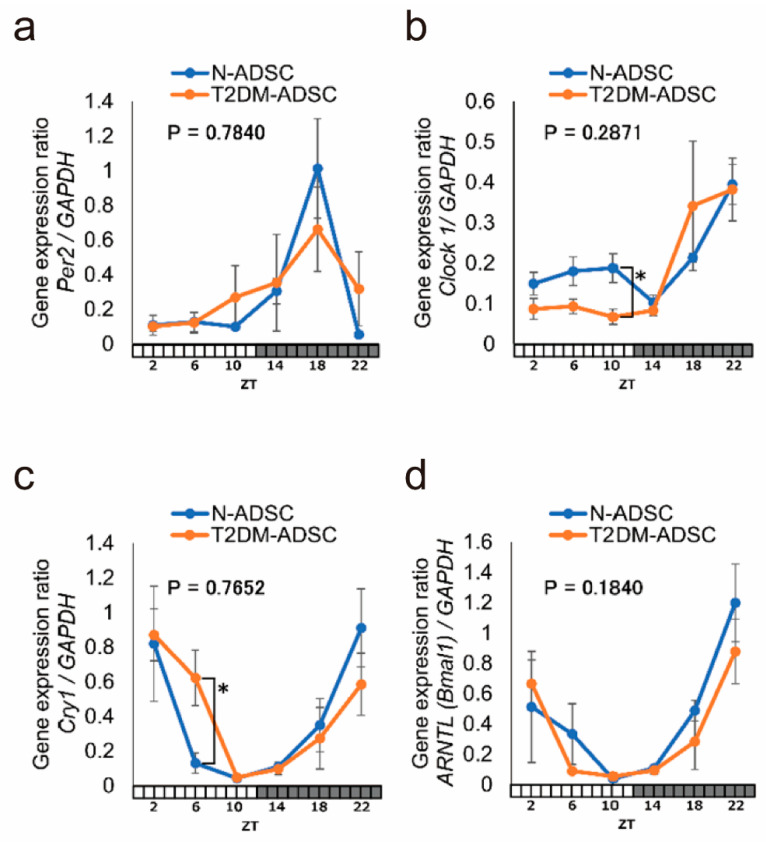
**Daily fluctuations in clock gene expression in adipose-derived mesenchymal stem cell transplants derived from a healthy individual and a patient with type 2 diabetes mellitus were measured using quantitative polymerase chain reaction**. N-ADSCs are shown as blue lines and T2DM-ADSCs as orange lines. (**a**) Daily fluctuations in *PER2* gene expression. (**b**) Daily fluctuations in *CLOCK1* gene expression. (**c**) Daily fluctuations in the *CRY1* gene expression. (**d**) Daily fluctuations in *ARNTL* (*BMAL1*) gene expression. * *p*-values < 0.05. Data are shown as the mean ± SD, n = 6 for each sampling point, normally distributed, *two-way ANOVA* with *Tukey–Kramer* post hoc test.

Next, quantitative PCR was performed to analyze daily fluctuations in the clock genes *PER2*, *CLOCK1*, *CRY1*, and *ARNTL* (*BMAL1*) (Figure 3a–d). These genes exhibited similar daily fluctuations in terms of phase and amplitude in both transplants (Figure 3a–d). Significant differences in expression were observed only in *CLOCK1* at ZT10 and *CRY1* at ZT6. At ZT6, the expression of *CRY1* was significantly increased in the T2DM-ADSC transplants compared with the N-ADSC transplants (Figure 3c). At ZT10, the expression of *CLOCK1* was significantly decreased in the T2DM-ADSC transplants compared with the N-ADSC transplants (Figure 3b).

Next-generation sequencing detected significant differences between the N-ADSCs and T2DM-ADSCs in the following categories related to tissue regeneration that are important for stem cell function: cell morphology, embryonic development, hair and skin development and function, organ development, organismal development, and tissue development (Table 1).

**Figure 4:**
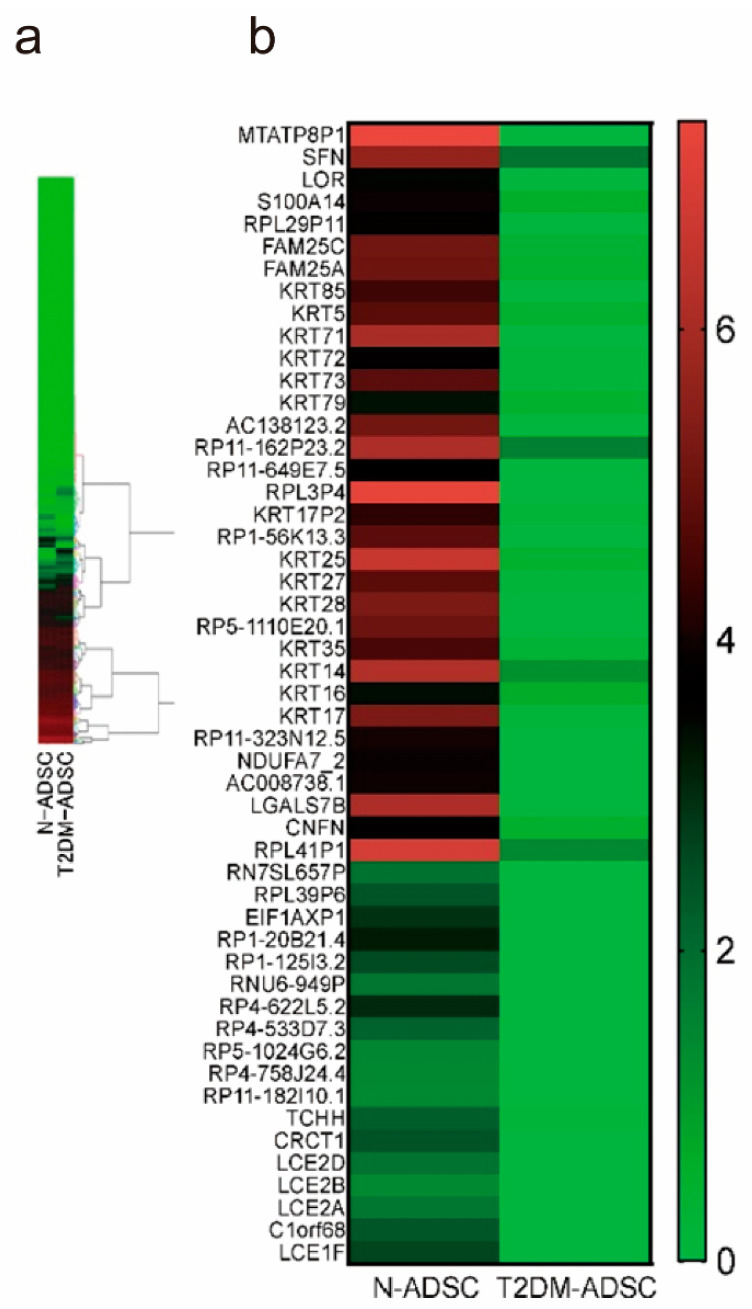
**Heat map of gene expression fluctuations in stem cell transplants.** (**a**) Heat map of gene expression fluctuations in transplant adipose-derived mesenchymal stem cells derived from a healthy individual and a patient with type 2 diabetes mellitus at Zeitgeber time 6 in the light period measured via next-generation sequencing. (**b**) Heat map of genes that exhibited a reduction in expression in transplant adipose-derived mesenchymal stem cells derived from a patient with type 2 diabetes mellitus compared with N-ADSC transplants.

The genes that exhibited variation in the expression between the N-ADSCs and T2DM-ADSCs were categorized based on next-generation sequencing (Figure 4a). Genes with considerably decreased expression in the T2DM-ADSC transplants were extracted as 17 candidate genes (Figure 4b). The expression of mitochondrially encoded ATP synthase 8 pseudogene 1 (*MTATP8P1*) and NADH:ubiquinone oxidoreductase subunit A7_2 (*NDUFA7_2*) varied between the light and dark periods.

**Figure 5:**
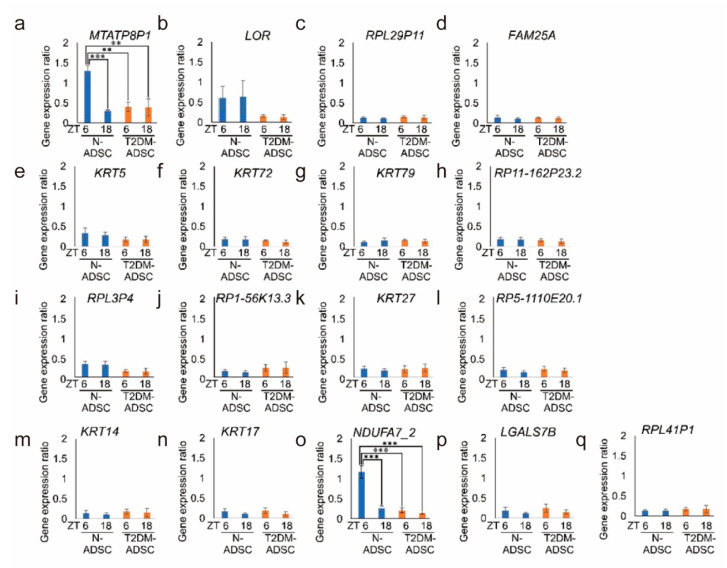
Gene expression was measured at two time points (Zeitgeber time 6 in the light period and Zeitgeber time 18 in the dark period). Gene expression of (**a**) *MTATP8P1*, (**b**) *LOR*, (**c**) *RPL29P11*, (**d**) *FAM25A*, (**e**) *KRT5*, (**f**) *KRT72*, (**g**) *KRT79*, (**h**) *RP11-162P23.2*, (**i**) *RPL3P4*, (**j**) *RP1-56K13.3*, (**k**) *KRT27*, (**l**) *RP5-1110E20.1*, (**m**) *KRT14*, (**n**) *KRT17*, (**o**) *NDUFA7_2*, (**p**) *LGALS7B*, and (**q**) *RPL41P1*. (**a**–**q**) The experiments used two types of ADSCs (N-ADSC-1 and -2 as two types of healthy individuals, T2DM-ADSC-1 and -2 as two types of T2DM patients) for each target gene. ** *p*-values < 0.01. *** *p*-values < 0.005. Data are shown as the mean ± SD, n = 4 for each sampling point, normally distributed, *two-way ANOVA* with *Holm* post hoc test.

The expression levels of these 17 genes were analyzed at two time points (ZT6 and ZT18 in the light and dark periods, respectively). In the N-ADSC transplant, two genes (*MTATP8P1* and *NDUFA7_2*) showed significantly lower expression levels at ZT18 (dark period) versus ZT6 (light period). In contrast, significant variations were not recorded in the T2DM transplant (Figure 5a,o). The remaining 15 genes did not show any significant variations in expression between the two time points (Figure 5b–n,p,q). The *MTATP8P1* and *NDUFA7_2* genes that showed variations between the two time points were related to mitochondria.

**Figure 6:**
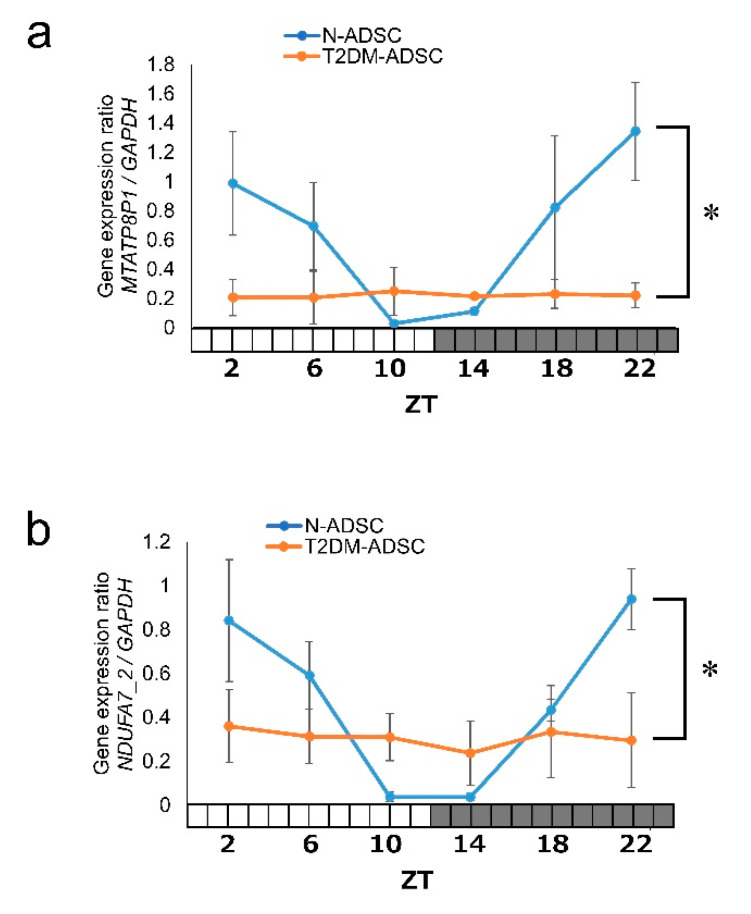
**Daily fluctuations in *MTATP8P1* and *NDUFA7_2* genes in T2DM-ADSCs.** N-ADSCs are shown as blue lines, and T2DM-ADSCs are shown as orange lines. The evaluation of (**a**) *MTATP8P1* and (**b**) *NDUFA7_2* on daily fluctuations in N-ADSCs and T2DM-ADSCs using RT-qPCR. Gene expressions were measured at six time points (Zeitgeber time 2, 6, and 10 in the light period and Zeitgeber time 14, 18, and 22 in the dark period). * *p*-values < 0.05. Data are shown as the mean ± SD, n = 6 for each sampling point, normally distributed, *two-way ANOVA* with *Tukey–Kramer* post hoc test.

The circadian rhythms of MTATP8P1 and NDUFA7_2 at six time points (ZT2, 6, 10, and ZT14, 18, and 22 in light and dark periods, respectively) were evaluated. Compared to N-ADSCs, the circadian rhythms of MTATP8P1 (Figure 6a) and NDUFA7_2 (Figure 6b) gene expression were lost in the T2DM-ADSCs.

## 3. Discussion

This study investigated the circadian rhythms of clock genes after stem cell transplantation. The results revealed the daily fluctuations in clock genes after the transplantation of N-ADSCs and T2DM-ADSCs. These fluctuations were similar in phase and amplitude between the two groups. The data demonstrated that the clock genes in stem cells were synchronized with those in living organisms.

In this study, the genes *MTATP8P1* and *NDUFA7_2* were identified to exhibit variations between two time points (ZT6 and ZT18). *MTATP8P1* is a factor involved in mitochondrial ATP synthesis-coupled proton transport [27] and periodic paralysis associated with cardiac myopathy and neuropathy [28,29]. *NDUFA7_2* is a gene encoding a subunit of NADH:ubiquinone oxidoreductase (complex I), which is present in the mitochondrial inner membrane [30]. Complex I regulates electron migration in the respiratory chain mediated by NADH [30]. This is the first study to report the occurrence of circadian rhythm fluctuations of MTATP8P1 and NDUFA7_2 in transplanted T2DM-ADSCs. The association between MTATP8P1 and NDUFA7_2 genes in ADSCs may be a molecular mechanism by which electron transfer by NDUFA7_2 promotes ATP transport by MTATP8P1. As a result, energy metabolism in N-ADSCs is considered to function normally. On the other hand, in T2DM-ADSCs, the circadian rhythmic control of ATP transport by NDUFA7_2 and MTATP8P1 is disrupted, which may lead to abnormalities in energy metabolism. We discuss the physiological significance of the loss of circadian rhythmicity of MTATP8P1 and NDUFA7_2 in the T2DM-ADSC transplantation groups. Mitochondria are responsible for energy production, and diurnal variations in energy production are observed between the active and resting phases of an individual. Loss of circadian rhythmicity of MTATP8P1 and NDUFA7_2 may lead to the aggravation of T2DM.

The validity and limitations of the methods utilized in this study are discussed below. Firstly, some clock genes exhibited rhythms, while others did not. The results of next-generation sequencing showed that the N-ADSC group had daily fluctuations in the clock genes *PER1*, *PER2*, *PER3*, *CRY1*, *CRY2*, *and ARNTL* (*BMAL1*), whereas daily fluctuations were not noted in *CLOCK1*, *CLOCK2*, *CLOCK3*, and *CLOCK4* (Figure 2a). Similarly, in the T2DM-ADSC group, *PER1*, *PER2*, *PER3*, *CRY1*, *CRY2*, and *ARNTL* (*BMAL1*) showed daily fluctuations, whereas daily fluctuations were not noted for *CLOCK1*, *CLOCK2*, *CLOCK3*, and *CLOCK4* (Figure 2b). In addition to the limit in the sensitivity of next-generation sequencing, the transcriptional regulatory sequence upstream of the clock genes may have been responsible for these differences. In mammals, basic helix-loop-helix protein (bHLH) transcription factors CLOCK and BMAL1 form a heterodimer, which binds to an E-box in the promoter regions of negative factor genes, such as *PER* and *CRY* [31,32]. This binding positively regulates the expression of genes, including *PER* and *CRY* [33]. Subsequently, the PER/CRY complex deactivates the transcriptional activity via CLOCK/BMAL1, thereby suppressing its own transcription [33]. This repetition forms an approximately 24 h cycle. In addition to this feedback loop, the responsive sequence of the clock genes and the secondary loop mediated by the Rev-responsive element and a D-box are functioning [32,33]. Given its limited detection sensitivity, it is impracticable to evaluate the circadian rhythms of the clock genes solely through next-generation sequencing. In this study, we performed an evaluation using quantitative PCR. The results revealed that the circadian rhythms of the clock genes *PER2*, *CLOCK*, *CRY1*, and *BMAL1* were similar between the N-ADSC and T2DM-ADSC groups.

The circadian rhythms of the clock genes are regulated at an approximately 24 h cycle by the central clock genes in the SCN [33]. Regulation by the central clock is abolished following tissue extraction and cell proliferation by primary culture. Accordingly, the clock genes fail to produce synchronized rhythms unless shocks are induced with high-concentration serum or dexamethasone [34]. In this study, we evaluated the impact of extraction time without inducing such shocks. Therefore, changes were likely smaller than those observed aftershocks induced by high-concentration serum or dexamethasone. However, an advantage of this evaluation system is the ability to detect differences based purely on the extraction time.

The limitations of this study are as follows. In this study, human adipose-derived ADSCs were transplanted into immunodeficient mice, so some of the results may not correlate with stem cell transplantation in humans. In particular, signaling from the SCN [35], a core clock gene, is preserved in this mouse model. However, since it is currently difficult to monitor clock genes in human transplanted tissues, the results of this study are useful for predicting human circadian rhythms after transplantation. Therefore, the results of this study have the potential to impact stem cell therapy in diabetes and other diseases, as the circadian rhythm of stem cells may be an indicator of transplantation efficacy. The loss of circadian variation in MTATP8P1 and NDUFA7_2 genes may be a useful marker of disruption of stem cell energy metabolism.

These findings may help to clarify the daily fluctuations in clock genes following stem cell transplantation. The *MTATP8P1* and *NDUFA7_2* genes, which showed variation between the two time points, are associated with mitochondria and should be noted for diurnal variation in mitochondrial genes. In future studies, we intend to clarify the molecular regulatory mechanism underlying the circadian variations in mitochondrial genes and functions in T2DM-ADSCs. Finally, we also wish to elucidate the circadian rhythms of mitochondria following stem cell transplantation in humans.

## 4. Methods

**ADSC transplantation model**

The ADSC transplantation model used in this study was established in our preceding studies [18,19]. N-ADSCs (PT-5006, Lonza, Basel, Switzerland) and T2DM ADSCs (PT-5007, Lonza) were seeded on 10 cm^2^ Petri dishes filled with 12 mL of medium containing cell growth factors (PT-4505, Lonza). The ADSCs were stripped using 3 mL of trypsin and EDTA solution (25300-062, Gibco, New York, NY, USA). N-ADSCs and T2DM ADSCs were cultured to form spheroids 1 week before the transplantation by adding cell growth factors supplied with the Matrigel matrix (356255, Corning, Somerville, MA, USA) for spheroid culture. The stem cell spheroids were mixed with the Matrigel matrix (354234, Corning) at a ratio of 1:1. The mixture containing 10^7^ ADSCs was filled in a syringe on ice and transplanted in the subcutaneous back tissues of immunodeficient mice (BALB/cAJcl-*nu*/*nu*). Three weeks after the transplantation, the skin grafts were removed for measurement. Five-week-old female BALB/cAJCL-*nu*/*nu* mice were purchased from CLEA Japan Incorporated (Tokyo, Japan) and raised under normal breeding conditions with a 12 h light/12 h dark cycle. To analyze the daily fluctuations in clock genes, the lighting start time (start point of the light period) was regarded as ZT0 (signifying 0 h in Zeitgeber time). Stem cell transplants were extracted at 4 h intervals from ZT2.

N-ADSCs, which were originally established via a primary culture of adipose tissue extracted from a 23-year-old healthy female without any underlying conditions, were purchased from LONZA KK (Cat numbers PT-5006, lot number 18TL212639). T2DM-ADSCs, which were originally established via a primary culture of adipose tissue extracted from a 39-year-old female with T2DM, were also purchased from LONZA KK (Cat numbers PT-5008, lot number 1F4619). Samples were collected 1 week after transplantation and used in the experiments.

This study was conducted according to the guidelines for the Proper Conduct of Animal Experiments and Related Activities in Academic Research Institutions and approved by the Institutional Review Board of Sanyo-Onoda City University (protocol code 2020-8-A, and date of approval 12 March 2020). This study was also conducted according to the ARRIVE guidelines.

This study used ADSCs that were ethically approved by Lonza. The ADSC consent form was obtained by Lonza, from which the cells were purchased.

**Next-generation sequencing**

Total RNA was extracted from the collected transplants using an RNeasy Mini Kit (74136, QIAGEN, Tokyo, Japan). Poly(A) RNA was extracted from 100 ng of total RNA and fragmented using the NEBNext Poly(A) mRNA Magnetic Isolation Module (E7490, NEB) and the NEBNext Ultra II RNA Library Prep Kit for Illumina (E7770, NEB). The fragmented poly(A) RNA was subjected to reverse transcription for the preparation of cDNA using the NEBNext First Strand Synthesis Enzyme Mix of the NEBNext Ultra II RNA Library Prep Kit for Illumina. Subsequently, the NEBNext Adaptor was added. The prepared cDNA was amplified via polymerase chain reaction (PCR) to create a library. The cDNA domain (75 bp) and barcode sequence were analyzed with fragment analysis using the Illumina NextSeq (550, NEB).

**Quantitative PCR**

The collected transplants were homogenized, and mRNA was extracted using an RNeasy Mini Kit. Reverse transcription was performed using a PrimeScript^®^ RT reagent Kit (RR037A, Takara Bio, Shiga, Japan) to prepare template cDNA. Primers were purchased from the TAKARA Perfect Real Time Primer Support System. The primers used are listed in Table 2. The template cDNA, PCR enzyme (SYBR Premix Ex Taq^TM^ II) (RR820A, Takara Bio), and forward and reverse primers were mixed for the subsequent reaction. The reaction was performed using a quantitative PCR device (StepOne-Plus-01, 4376592, Applied Biosystems, Foster City, CA, USA). In real-time PCR, the conditions were 95 °C for 30 s (Step 1), followed by 95 °C for 5 s and 60 °C for 60 s (Step 2) for 40 cycles. The expression levels of target genes were calculated using calibration curves, and the relative expression was calculated using glyceraldehyde-3-phosphate dehydrogenase (GAPDH) as an internal standard.

**Statistical analysis**

Results are presented as mean ± standard deviation values. A comparison between multiple groups was performed using a *two-way ANOVA* with *Tukey–Kramer* post hoc tests or *Holm* post hoc tests, with *p*-values < 0.05 denoting statistical significance.

## Figures and Tables

**Table 1:** Genes that showed greater than 2-fold changes in N-ADSCs and T2DM-ADSCs were classified using The Database for Annotation, Visualization, and Integrated Discovery (DAVID). Categories that were hit in the Kyoto Encyclopedia of Genes and Genomes (KEGG) were identified according to *p*-value. The top six categories of the fluctuating genes in adipose-derived mesenchymal stem cells derived from a healthy individual and a patient with type 2 diabetes mellitus transplant were determined via next-generation sequencing.

Category	*p*-Value	Molecules
**Cell Morphology**	2.88 × 10^−49^–2.98 × 10^−2^	EPCAM, KRT1, KRT12, KRT14, KRT15, KRT16, KRT17, KRT25, KRT26, KRT27, KRT28, KRT31, KRT32, KRT33A, KRT35, KRT5, KRT6A, KRT6B, KRT71, KRT72, KRT73, KRT74, KRT75, KRT76, KRT79, KRT85, KRT86, KRTAP2-1, LORICRIN, mir-199, MSX2, PKP1, SERPINB5, SFN, SP6, SPINK5, SPRR1A, SPRR1B, ST14, TCHH, TYRP1
**Embryonic Development**	2.3 × 10^−43^–3.75 × 10^−2^	COL10A1, DCT, EPCAM, HOXC13, KRT1, KRT12, KRT14, KRT15, KRT16, KRT17, KRT25, KRT26, KRT27, KRT28, KRT31, KRT32, KRT33A, KRT35, KRT5, KRT6A, KRT6B, KRT71, KRT72, KRT73, KRT74, KRT75, KRT76, KRT79, KRT85, KRT86, KRTAP2-1, LHX2, LORICRIN, mir-27, MSX2, PKP1, SERPINB5, SFN, SOX21, SP6, SPINK5, SPRR1A, SPRR1B, ST14, TFAP2A, TFAP2B, TYRP1
**Hair and Skin Development and Function**	2.3 × 10^−43^–3.71 × 10^−2^	DCT, EPCAM, HOXC13, KRT1, KRT12, KRT14, KRT15, KRT16, KRT17, KRT25, KRT26, KRT27, KRT28, KRT31, KRT32, KRT33A, KRT35, KRT5, KRT6A, KRT6B, KRT71, KRT72, KRT73, KRT74, KRT75, KRT76, KRT79, KRT85, KRT86, KRTAP2-1, LGALS7/LGALS7B, LHX2, LORICRIN, mir-27, MSX2, PKP1, SFN, SOX21, SP6, SPINK5, SPRR1A, SPRR1B, ST14, TFAP2A, TYRP1
**Organ Development**	2.3 × 10^−43^–3.62 × 10^−2^	COL10A1, DCT, HOXC13, KRT1, KRT12, KRT14, KRT15, KRT16, KRT17, KRT25, KRT26, KRT27, KRT28, KRT31, KRT32, KRT33A, KRT35, KRT5, KRT6A, KRT6B, KRT71, KRT72, KRT73, KRT74, KRT75, KRT76, KRT79, KRT85, KRT86, KRTAP2-1, LGALS7/LGALS7B, LHX2, LORICRIN, mir-199, mir-27, MSX2, PKP1, SFN, SOX21, SP6, SPINK5, SPRR1A, SPRR1B, ST14, TFAP2A, TFAP2B, TYRP1
**Organismal Development**	2.3 × 10^−43^–4.01 × 10^−2^	COL10A1, DCT, EPCAM, ESRP1, GPR87, HOXC13, KRT1, KRT12, KRT14, KRT15, KRT16, KRT17, KRT25, KRT26, KRT27, KRT28, KRT31, KRT32, KRT33A, KRT35, KRT5, KRT6A, KRT6B, KRT71, KRT72, KRT73, KRT74, KRT75, KRT76, KRT79, KRT85, KRT86, KRTAP2-1, LHX2, LORICRIN, mir-199, mir-27, MSX2, PKP1, RAB25, SERPINB5, SFN, SOX21, SP6, SPINK5, SPRR1A, SPRR1B, ST14, TFAP2A, TFAP2B, TYRP1
**Tissue Development**	2.3 × 10^−43^–4.01 × 10^−2^	COL10A1, DCT, EPCAM, ESRP1, GPR87, HOXC13, KRT1, KRT12, KRT14, KRT15, KRT16, KRT17, KRT25, KRT26, KRT27, KRT28, KRT31, KRT32, KRT33A, KRT35, KRT5, KRT6A, KRT6B, KRT71, KRT72, KRT73, KRT74, KRT75, KRT76, KRT79, KRT85, KRT86, KRTAP2-1, LGALS7/LGALS7B, LHX2, LORICRIN, mir-27, MSX2, PKP1, RAB25, SCT, SERPINB5, SFN, SOX21, SP6, SPINK5, SPRR1A, SPRR1B, ST14, TFAP2A, TFAP2B, TYRP1

**Table 2:** The primers list for circadian clock gene.

**Forward primer**					
**Gene Symbol**	**Acc. No.**	**Position**	**Forward**	**GC%**	**Tm**
**GAPDH**	NM_002046.4	415	GCACCGTCAAGGCTGAGAAC	60	63.3
**Per2**	NM_022817.2	4929	CGTTGGAACCACCCAGACATC	57.1	64.9
**CLOCK**	NM_004898.2	5092	CCCACATTCCCATGAATATCAAGA	41.7	64
**Cry1**	NM_004075.3	2458	AATATCCCAGGTTGTAGCAGCAGTG	48	64.6
**Bmal1**	NM_001178.4	2134	GCCTACTATCAGGCCAGGCTCA	59.1	65
**Reverse primer**					
**Gene Symbol**	**Acc. No.**		**Reverse**	**GC%**	**Tm**
**GAPDH**	NM_002046.4	415	TGGTGAAGACGCCAGTGGA	57.9	64
**Per2**	NM_022817.2	4929	ATGCAGTCGCAAGCTGTCAGA	52.4	64.5
**CLOCK**	NM_004898.2	5092	GCTATTGCCATATTCCACAACATCC	44	64.6
**Cry1**	NM_004075.3	2458	GTTTGCTGACTGTCGCCATGA	52.4	64.6
**Bmal1**	NM_001178.4	2134	AGCCATTGCTGCCTCATCATTAC	47.8	64.6

## Data Availability

The datasets generated during the current study are available in the Harvard Dataverse repository at https://doi.org/10.7910/DVN/DWOLNR (accessed on 7 July 2023).
